# The MicroRNA397a-LACCASE17 module regulates lignin biosynthesis in *Medicago ruthenica* (L.)

**DOI:** 10.3389/fpls.2022.978515

**Published:** 2022-08-18

**Authors:** Yutong Zhang, Xiaotong Shan, Qiao Zhao, Fengling Shi

**Affiliations:** ^1^Key Laboratory of Forage Cultivation, Processing and High Efficient Utilization of the Ministry of Agriculture and Key Laboratory of Grassland Resources of the Ministry of Education, College of Grassland Resources and Environment, Inner Mongolia Agricultural University, Hohhot, China; ^2^Shenzhen Key Laboratory of Synthetic Genomics, Guangdong Provincial Key Laboratory of Synthetic Genomics, CAS Key Laboratory of Quantitative Engineering Biology, Shenzhen Institute of Synthetic Biology, Shenzhen Institutes of Advanced Technology, Chinese Academy of Sciences, Shenzhen, China

**Keywords:** *Medicago ruthenica*, secondary cell wall, lignin, LACCASE17, MicroRNA397a

## Abstract

Mechanical strength is essential for the upright growth habit, which is one of the most important characteristics of terrestrial plants. Lignin, a phenylpropanoid-derived polymer mainly present in secondary cell walls plays critical role in providing mechanical support. Here, we report that the prostrate-stem cultivar of the legume forage *Medicago ruthenica* cultivar ‘Mengnong No. 1’ shows compromised mechanical strength compared with the erect-stem cultivar ‘Zhilixing’. The erect-stem cultivar, ‘Zhilixing’ has significantly higher lignin content, leading to higher mechanical strength than the prostrate-stem cultivar. The low abundance of miRNA397a in the Zhiixing cultivar causes reduced cleavage of *MrLAC17* transcript, which results in enhanced expression level of *MrLAC17* compared to that in the prostrate-stem cultivar Mengnong No. 1. Complementation of the *Arabidopsis lac4 lac17* double mutants with *MrLAC17* restored the lignin content to wild-type levels, confirming that MrLAC17 perform an exchangeable role with *Arabidopsis* laccases. *LAC17-*mediated lignin polymerization is therefore increased in the ‘Zhilixing’, causing the erect stem phenotype. Our data reveal the importance of the miR397a in the lignin biosynthesis and suggest a strategy for molecular breeding targeting plant architecture in legume forage.

## Introduction

*Medicago ruthenica* (L.) is an allogamous, diploid (2n = 2x = 16) perennial legume forage of the *Medicago* genus (Small and Jomphe, 1989). Compared to other *Medicago* species, *M. ruthenica* has enhanced tolerance to environmental stresses, and making it a valuable resource for the resistance breeding of *Medicago sativa* (alfalfa) (Wang D. K. et al., 2008; Guan et al., 2009; Yang et al., 2011). Due to its adaptability to harsh environments, *M. ruthenica* is widely distributed in northern China, particularly in the Mongolian Plateau, where it is an important forage legume in cultivated and natural grasslands. *M. ruthenica* is related to *M. sativa*, which is one of the most important forage legume worldwide (Campbell et al., 1997, 1999). Therefore, *M. ruthenica* is believed to be a useful genetic model to elucidate mechanisms underlying tolerance to environmental stress, knowledge of which could be used to improve traits of alfalfa. Recent published genome information provides a powerful tool to investigate mechanisms underlying important agronomic traits of *M. ruthenica* (Wang et al., 2021).

Unlike other *Medicago* species, *M. ruthenica* does not have an upright growth habit, and its architecture is poorly suited to modern cultivation and harvesting systems, and it can only be utilized through grazing (Campbell et al., 1997). To improve this plant’s architectural characteristics, a cultivar with erect stems was bred successfully by cultivating and domesticating wild germplasm from different regions, and an erect cultivar was formally registered in 1993 (Wu et al., 1993). However, the detailed molecular mechanisms underlying the enhanced stem mechanical support have not previously been elucidated.

The upright growth habit of higher terrestrial plants is conferred by the deposition of secondary cell wall in fiber and xylem vessels following cell growth. Secondary cell walls mainly comprises lignin, cellulose, and hemicellulose which provide mechanical strength to support upright growth (Somerville et al., 2004). It has been reported that plants with disruption of the genes participating in secondary cell wall formation show reduced mechanical strength (Zhang et al., 2018). Disrupted secondary cell wall generally leads to lodging or prostrate stem phenotypes, which is disadvantageous for crop production (Zhong et al., 2009; Zhang and Zhou, 2011).

Lignin, a major component of the plant secondary cell wall, is a complex phenolic polymer composed of three *p*-hydroxycinnamyl alcohol monolignols: *p*-coumaryl, coniferyl, and sinapyl alcohols (Boerjan et al., 2003). Lignin polymers are mainly deposited in the cellulose framework, providing rigidity and strength for plants to stand upright and endowing conducting tissue with hydrophobicity (Vanholme et al., 2008; Weng and Chappie, 2010). Monolignol is synthesized in the cytoplasm and then transferred to the cell wall where it is oxidized into the polymers by laccases (and/or peroxidases) (Vanholme et al., 2008, 2010; Bonawitz and Chapple, 2010). Laccases are the largest component of multi-copper oxidoreductases family. Plant laccase was the first enzyme reported to be able to polymerize lignin monomers *in vitro* (Freudenberg, 1959). The role of laccase in stem lignification has been clearly demonstrated by reverse genetics. For example, overexpression of the cotton laccase (*GaLAC1*), in *Populus* resulted in increased lignin content, and total lignin in transgenic plants increased by up to 20%, indicating that laccases are involved in lignin polymerization (Wang J. et al., 2008). In *Arabidopsis*, simultaneous disruption of *LAC4* and *LAC17* resulted in reduced lignin content in the stem, and the *lac4 lac17 lac11* triple mutant resulted in severe retardation of plant growth and vascular development (Berthet et al., 2011; Zhao et al., 2013). A recent study showed that laccases play a key role in the spatially organized polymerization of H-lignin and G-lignin during compression wood formation (Hiraide et al., 2021). Furthermore, the expression level of laccase genes are controlled by both transcription and post-transcription regulation. MYB63 and MYB58 have been identified as transcriptional activators regulating lignin biosynthesis, and MYB58 directly activates *LAC4* in *Arabidopsis* (Zhou et al., 2009). miR397 is a family of small and non-coding microRNAs (miRNAs) conserved in dicots, monocots, and gymnosperms (Kozomara and Griffiths-Jones, 2011), and the miR397 has been identified to directly target laccase transcripts in *Arabidopsis* (Jones-Rhoades and Bartel, 2004), *Populus trichocarpa* (Lu et al., 2008) and Rice (Zhang Y. C. et al., 2013). In *Arabidopsis*, overexpression of miR397b was found to reduce lignin deposition and overexpression of miR397b-resistant laccase mRNA results in increased lignin formation (Wang et al., 2014). In the model woody plant, *P. trichocarpa*, miR397a has been validated to be a master regulator of lignin polymerization (Lu et al., 2008). In rice, miR397-laccase gene regulation module also can change lignification, facilitating stems erect growth (Swetha et al., 2018). In addition, miR408 and miR857 (in *Arabidopsis*)/miR528 (in rice) have also been reported to target plant laccases or other blue copper oxidases (Abdel-Ghany and Pilon, 2008; Li et al., 2010).

Two cultivars of *M. ruthenica* with contrasting stem habits were used in the present research, an erect cultivar ‘Zhilixing’ and a prostrate cultivar ‘Mengnong No.1’. The mechanisms related to differences in lignin synthesis in the two cultivars were investigated by metabolite and transcriptome analysis. Differentially expressed genes (DEGs) identified by functional classification and metabolic pathways were further validated using quantitative real-time PCR (qRT-PCR). We found that *MrLAC17* transcription levels differed between the two cultivars, whereas transcript levels of other monolignol biosynthetic genes remained unchanged. Our final objective was to identify important factors that impact the difference in expression of *MrLAC17* in lignin synthesis between the two cultivars. *MrLAC17* was overexpressed in *Arabidopsis thaliana* and its function was characterized in this study. The differences in expression of miR397a in the two cultivars were confirmed by analysis of the miRNAs database of *M. ruthenica* stems, and *MrLAC17* was identified as the target of mr-miR397a in *M. ruthenica* by bioinformatic analysis. We conclude that the miR397a-*LAC17* module regulates lignin content in erect and prostrate stems of *M. ruthenica*.

## Materials and methods

### Plant materials and sampling site

Two *M. ruthenica* cultivars were used in this study: Zhilixing, an erect-stem cultivar, and Mengnnog No. 1, a prostrate-stem cultivar. Both cultivars were bred by Inner Mongolia Agricultural University (IMAU), China. The erect-stem cultivar [*M. ruthenica* (L.) Sojak cv. ‘Zhilixing’] was approved by the Chinese Herbage Varietal Resources Registration Board in 1993 and registered as a new variety (Registration No. 130), and the prostrate-stem cultivar [*M. ruthenica* (L.) Trautv. ‘Mengnong No.1’] was approved by the Inner Mongolia Autonomous Region Herbage Varietal Resources Registration Board in 2019 and registered as a new variety (Registration No.: Inner Mongolia Approval-003-2019). Two other prostrate *M. ruthenica* accessions, ‘Mengnong No.3’ and wild-type germplasm materials, were also used in this study. ‘Mengnong No.3’ was also bred by IMAU, and the wild-type seeds were collected from Xinghe County, Inner Mongolia Autonomous Region, China. Before the experiments were conducted, all plant accessions were cultivated in Science and Technology Park of IMAU (111°23′46″E, 40°31′17″N) under normal management conditions for more than 10 years, during which the phenotypic traits remained stable in the meantime. Seeds of all accessions seeds were sown at the experimental base of IMAU, located in Hohhot City, Inner Mongolia, China (111°22′30″E, 40°41′30″N) at the end of August, 2018. The middle stems (internodes 4–6) of the Zhilixing and Mengnong No.1 cultivars, as well as the other two accessions, were collected at the early branching stage in May 2019. All samples were immediately frozen in liquid nitrogen and stored at −80°C for subsequent RNA or DNA extraction.

The *Arabidopsis thaliana* ecotype Columbia (Col-0), and *Nicotiana benthamiana* plants were grown in a greenhouse (22°C, 16 h light/8 h dark). The *lac4* mutant GabiKat-720G02 and the *lac17* mutant Salk_016748 were provided by Dr. Qiao Zhao (Shenzhen Institutes of Advanced Technology, Chinese Academy of Sciences, China).

### Phenotype analyses

Plant type indexes, including plant height and stem diameter, were measured at the early branching stage in May. The natural and absolute plant height were measured using a ruler, and the plant type coefficient was calculated by their ratio (plant type coefficient = natural height/absolute height). A plant type coefficient less than 0.5 indicated a prostrate stem, while a coefficient of more than 0.8 indicated an erect stem, while a coefficient of 0.5–0.7 was characterized as an oblique stem (Wang et al., 2013).

### Stem anatomical observations

The anatomical structure of stems was observed by paraffin sections. The fourth to sixth internodes excised from the two *M. ruthenica* cultivars were immobilized with an FAA solution (70% ethyl alcohol 90 mL, formaldehyde 5 mL, and glacial acetic acid 5 mL), and the sections were dehydrated thought xylenes and graded ethanol (100% I–100% II–75% alcohol). Each step took 5 min. The samples were dehydrated with a mixture of xylene: anhydrous alcohol (1:3, 1:1, 3:1, v/v) and then with a mixture of xylene:chloroform (9:1, v/v). Each step took 30 min. Subsequently, the samples were subjected to transparent treatment, paraffin-embedded, sectioned (RM2016, Shanghai Leica Instruments Co., Ltd.), dewaxing and safranin O-Fast green staining. Finally, the stained sections were observed under an optical microscope (Nikon Eclipse E100, Nikon, Japan). Paraffin sections prepared by the above methods were used to measure the length and width diameters, respectively.

### Cell wall component measurement

Carbohydrate analysis samples were collected from the target stems at the early branching stage and ball-milled into powders using a Tissuelyser II Mill (Qiagen, CA, United States) with the setting:60 s, 30 Hz, and passed through a 100-mesh sieve. The lignin content and composition of stem samples were determined using previously published thioacidolysis procedures (Zhao et al., 2010). Approximately 20 mg (± 0.1 mg) of extractive-free samples were reacted with 20 ml of 0.2 M BF3 etherate in a dioxane/ethanethiol (8.75:1) mixture. The monomers derived from lignin were identified by gas chromatography-mass spectrometry (GC/MS), and quantified by GC. The content of lignin in dry stems of transgenic *Arabidopsis thaliana* was measured by acetyl bromide using ∼100 mg (Hatfield et al., 1999). The monosaccharide and cellulose quantification were determined according to the method of Zhang and Zhou (2017). In brief, the samples were sequentially extracted using alcohols, treated with an amylase to obtain destarched cell wall residues. Two milligram cell wall residues of each replicate were hydrolyzed in 2 M trifluoroacetic acid. The lysate was reduced with 1 M sodium borohydride and further derivated with acetic anhydride. The generated alditol acetates were subjected to GC (7890B)-coupled mass spectrometry (5977A, Agilent) for quantification according to calibrated standard curves. The remaining pellets were subjected to cellulose quantification using the anthrone method.

### DNA and RNA extraction and cDNA synthesis

*M. ruthenica* stems were collected to extract the genomic DNA and total RNA using the Plant Genomic DNA Kit and RNAprep Pure Plant Plus Kit, respectively (both from TianGen Biotech., Beijing, China) following the manufacturer’s instructions. Reverse transcription using *EasyScript*^®^ One-Step gDNA Removal and cDNA Synthesis SuperMix Kit (TransGen Biotech., Beijing, China) with oligo (dT)18 primers for target mRNAs and specific stem-loop RT primers for miRNA.

### mRNA-seq and analysis

High-quality RNA of the two cultivars (three biological replicates of each cultivar) were sent to Lianchuan Biotechnology Co., Ltd. (Hangzhou, China) for cDNA library construction and RNA sequencing (RNA-seq). *M. ruthenica* genome information was not available at the beginning of this study, so the clean reads of six samples were combined to *de novo* assemble the transcriptome using the reference genome-independent Trinity method (Grabherr et al., 2011). The genome information of *M. ruthenica* (accession WNNG0000000)^1^ was published by Wang (Wang et al., 2021), and the previously obtained clean data of each sample were mapped to the reference genome to verify the reliability of the *de novo* assembly.

The expression of unigene was normalized to transcripts per million (TPM) values, and DEGs identification were performed among samples or groups using edgeR software with log2 (fold change) > 1 or log2 (fold change) < −1 and with statistical significance tested at *p*-value < 0.05 using the R package edgeR (Smyth, 2010). Next, Gene Ontology (GO)^2^ and Kyoto Encyclopedia of Genes and Genomes (KEGG)^3^ enrichment analyses were performed on all DEGs, and hypergeometric tests with *p* ≤ 0.05 as a threshold were used to identify the significant enrichment of GO terms and KEGG pathways.

### MicroRNA-seq and analysis

Six small RNA libraries were constructed by BGI Genomics (The Beijing Genomics Institute), three biological replicates of each cultivar. The sequencing of miRNAs was performed using the BGISEQ-500 technology. The clean reads were BLASTed against the miRNA database miRbase 22.1^4^ to identify the known miRNAs. The expression was calculated based on the TPM. We used the above-mentioned *M. ruthenica* genome data as a reference for analysis. Since miRNAs are mainly bound to targets by complementary pairing, transcriptome data are further used as the targets for matching mature miRNA sequences using TargetFinder and psRobot (Peer, 2010).

### Quantitative real-time PCR analysis

The qRT-PCR analyses were carried out using TB Green Premix ExTaq (TaKaRa, Dalian, China) and a CFX96 Manager Real-Time PCR software system (Bio-Rad, Hercules, CA, United States). Gene-specific primers for qRT-PCR were designed online,^5^ the *M. ruthenica* actin gene (GenBank: KF149988.1) was used as a standard control for analyzing the mRNA expression level. For the miRNA expression analysis, the U6 snRNA of *M. ruthenica* was used as an internal reference gene. Each sample (including three biological replicates) was quantified with three technical replicates. The 2^–△△CT^ method was used to calculate relative gene expression level (Livak and Schmittgen, 2001). All primers used in qRT-PCR are shown in Supplementary Table 1.

### Cloning of *MrLAC17* and sequences analysis

Specific primers were designed according to the predicted coding sequence (CDS) to amplify the full-length cDNA, and the amplified products were cloned into a *pEASY*-Blunt Zero cloning vector (Cat No. CB501, TransGen Biotech, Beijing, China) for sequencing confirmation. Following the Genome Walking Kit (Takara, Dalian, China) manufacturer’s instructions, the promoters of *MrLAC17* were cloned from genomic DNA of two *M. ruthenica* cultivars. The PCR products were sub-cloned into *pEASY*-T1 Cloning Vector and sequenced (Cat No. CT101, TransGen Biotech, Beijing, China). Finally, 1.0 kb upstreams of the ATG transcriptional start site were tentatively selected as promoter sequences. All primers used for cloning are shown in Supplementary Table 1. The PLANTCARE database^6^ and PLACE database^7^ were used to identify potential *cis*-regulatory elements within the promoter.

To perform homology comparisons, the full-length amino acid sequences of MrLAC17 protein was first compared with the homologs of laccases (or ascorbate oxidase) of other plant species using the default parameters given by Clustal Omega.^8^ The aligned sequences were further used to build a neighbor-joining tree using MEGA software (version 11.0) (Tamura et al., 2021). Database accession numbers of laccases (or ascorbate oxidase) were listed in Supplementary Table 2.

### Dual-luciferase assay

The transcriptional activation was performed in *Nicotiana benthamiana* leaves using dual-luciferase assay. The 1.0 kb promoters of *MrLAC17* were cloned into the pGreenII 0800-LUC vector to construct *P-LUC* reporter genes in the two *M. ruthenica* cultivars (Hellens et al., 2005). Each constructed vector was transformed into *Agrobacterium tumefaciens* strain GV3101, which contained the helper plasmid pSoup-P19 (Hellens et al., 2005). The full-length CDS (without stop codons) of AtMYB63 (At1g79180) and *GUS* were cloned into the pEarleyGate 101 vector transformed into *A. tumefaciens* strain GV3101. The suspension of *A. tumefaciens* in different combinations were seeped into *N. benthamiana* leaves, and the samples were collected after 48 h for the dual-luciferase assay. A Dual-Luciferase Reporter Assay System (Promega) was used to detect luciferase activities. Follow the manufacturer’s instructions for specific steps. Each sample contained three biological replicates. Primers for all constructs are listed in Supplementary Table 1.

### Complementation of the *Arabidopsis thaliana lac4 lac17* mutant

The 35S:MrLAC17-YFP construct was conducted using the ClonExpress II One Step Cloning Kit (catalog no. C112; Vazyme). The CDS of MrLAC17 (without stop codon) was amplified using the primers1300-MrLAC17-F and 1300-MrLAC17-R (Supplementary Table 1), and then cloned into a modified pCAMBIA1300 vector with YFP tag. The plasmid 35S:MrLAC17-YFP and empty vector (35S:YFP) were transferred into *lac4 lac17* and Col-0 plants using the floral dip method of *A. tumefaciens* GV3101 (Clough and Bent, 2010).

### Statistical analysis

All the data were subjected to statistical analysis with a Duncan test (Duncan, 1955) using SAS software (version 9.4).

## Results

### Zhilixing and Mengnong No.1 show significant differences in terms of stem morphological characteristics and secondary cell wall biosynthesis

**FIGURE 1:**
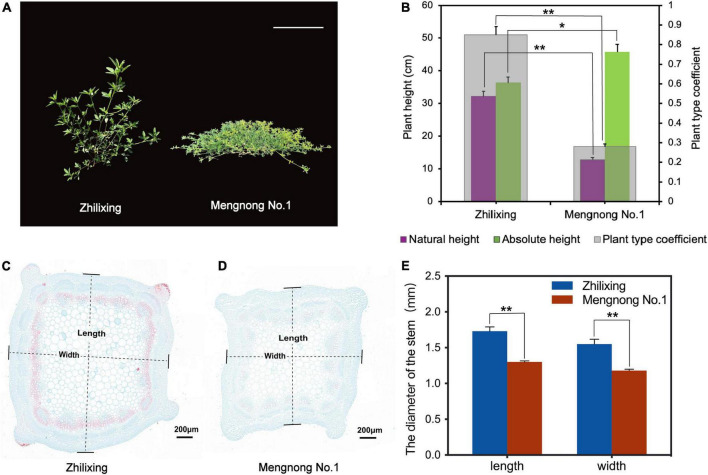
Phenotypic analysis of *M. ruthenica* cultivars Zhilixing and Mengnong No.1. **(A)** Two-month-old plants of the Zhilixing and the Mengnong No.1. Bar = 10 cm. **(B)** Comparison of the plant height (absolute plant height and natural plant height) and plant type coefficient between the two cultivars. The plant type coefficient was calculated by the ratio plant type coefficient = natural height/absolute height. A plant type coefficient less than 0.5 indicates a prostrate stem, greater than 0.8 indicates an erect stem, and 0.5–0.7 indicates an oblique stem. Values are means ± S.D. of fifteen biological replicates. **(C–E)** Stem cross section of the Zhilixing **(C)** and the Mengnong No.1 **(D)**. Lignified cells appear in red color. Stem (internodes 4–6) from the same period as **(A)** at 5.0x magnifications. Bar = 200 μm in **(C,D)**. **(E)** The stem diameter was measured according to the “length” and “width” of **(C,D)** labels. Values are means ± S.D. of six technical replicates (different paraffin sections) from three biological replicates. In **(B,E)**, asterisks indicate significant differences between the two cultivars, as determined by Student’s *t*-test (**p < 0.01, *P < 0.05).

Differences in plant architecture were very significant between the *M. ruthenica* cultivars Zhilixing and Mengnong No.1. The Zhilixing cultivar showed erect growth, whereas the cultivar Mengnong No.1 showed prostrate growth (Figure 1A). In forage grass, the ratio of natural plant height to absolute plant height (plant type coefficient) was used to represent stem type (Wang et al., 2013). Accordingly, the plant type coefficient of cv. Zhilixing was greater than 0.8 for erect stems, whereas the plant type coefficient of cv. Mengnong No.1 was less than 0.5 for prostrate stems (*p* < 0.01, Figure 1B). To investigate vascular development in the stem, two *M. ruthenica* cultivars stems were embedded in paraffin, and stained with Safranin O-Fast Green Staining. The stem cross-sections of *M. ruthenica* were examined under the microscope, and it was found that fewer cells in the stem tissues of Mengnong No.1 were lignified, whereas a large amount of lignification was observed in the stems of Zhilixing. Furthermore, the stem diameter of Zhilixing was significantly larger than that of Mengnong No.1 (*p* < 0.01, Figures 1C–E).

**FIGURE 2:**
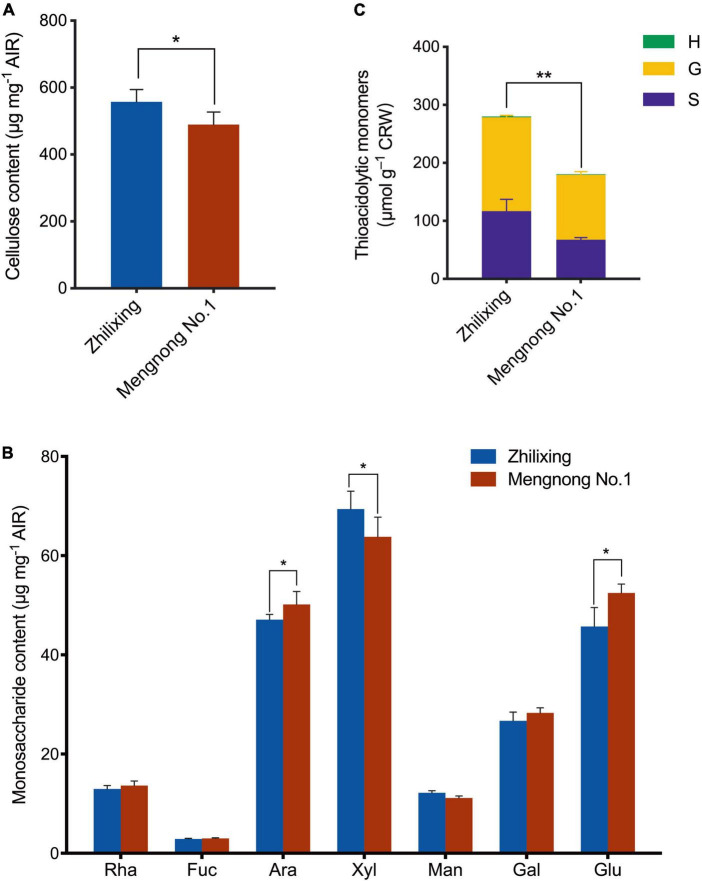
Measurement of cell wall composition in the *M. ruthenica* cultivars Zhilixing and Mengnong No.1. **(A,B)** Determination of polysaccharides content in the *M. ruthenica* cultivars Zhilixing and Mengnong No.1. **(A)**, cellulose content **(B)**, neutral monosaccharides (Rha, rhamnose; Fuc, Fucose; Ara, Arabinose; Xyl, Xylose; Man, Mannose; Gal, Galactose; Glu, Glucose). In **(A,B**), values are means ± S.D. of six biological replicates. Asterisks indicate significant differences between the two cultivars, as determined by Student’s *t*-test (*P < 0.05). **(C)** Determination of lignin composition and content in the two *M. ruthenica* cultivars. Values are means ± S.D. of six biological replicates. Asterisks indicate significant differences in G-, S-lignin and total lignin content between the two cultivars, as determined by Student’s *t*-test (**p < 0.01).

The difference in stem morphology between the two *M. ruthenica* cultivars suggested that the components of secondary cell wall may be altered. Therefore, we determined the contents of lignin and the major polysaccharides of the secondary cell wall in the stem of two cultivars. Compared with cv. Mengnong No.1, the contents of cellulose in cv. Zhilixing were significantly increased (*P* < 0.05, Figure 2A), whereas the little difference in monosaccharides content was found between the two cultivars (*P* < 0.05, Figure 2B). The lignin composition of the two *M. ruthenica* cultivars, including guaiacyl (G), syringyl (S), and *p*-hydroxyphenyl (H) units, was determined by the thioacidolysis method (Lapierre et al., 1985). In comparison with the Mengnong No.1, the G and S -type lignin content of the Zhilixing was significantly increased by nearly 40% (*p* < 0.01, Figure 2C). The H-type lignin content was very low in *M. ruthenica* and there was no significant difference between two *M. ruthenica* cultivars (*P* > 0.05).

### Identification and analysis of differentially expressed genes involved in cell wall-related biogenesis

**FIGURE 3:**
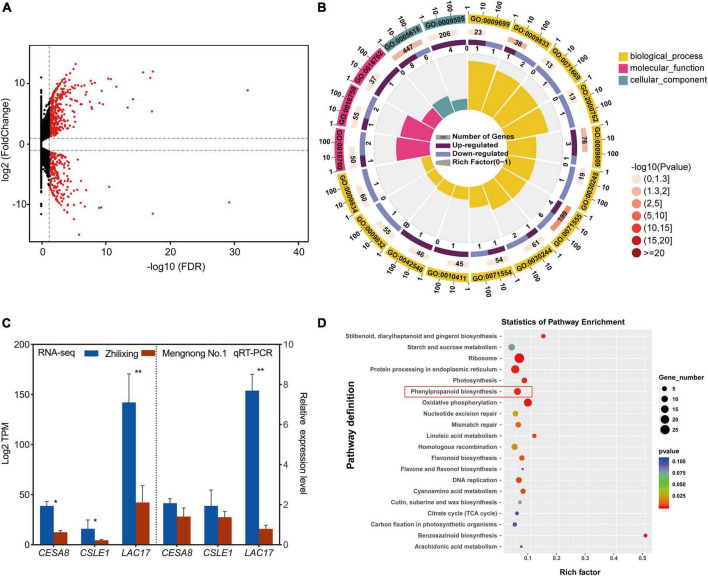
Differential expression of cell wall related genes in the *M. ruthenica* cultivars Zhilixing and Mengnong No.1. **(A)** MA plot (M-vs.-A plot) showing the relative expression level of all unigenes between the two cultivars. Red and black dots represent differentially expressed genes and genes with no significant expression difference, respectively. **(B)** GO categories associated with cell wall-related biogenesis. The outer circle presents the classification of enrichment, “1, 10, 100” is the coordinate ruler of the number of genes. The second circle (outside-to-inside, similarly hereinafter) gives the number of this classification in the background gene and the *Q*-value or *P*-value. The third circle is a bar chart showing the proportion of up-regulated DEGs and down-regulated DEGs (Mengnong No.1 vs. Zhilixing), and the specific values are shown below. The fourth circle gives a richness factor value of each classification (the number of DEGs divided by the number of background genes in the classification). **(C)** Expression of cell wall-related unigenes of the two *M. ruthenica* cultivars by RNA-Seq and qRT-PCR analysis. The expression level in Mengnong No.1 was set to 1. *MrActin* was used as the internal control. Values are means ± S.D. of three biological replicates. Asterisks indicate significant differences determined by Student’s *t*-tests (**p < 0.01, *P < 0.05). **(D)** KEGG enrichment analysis with the DEGs.

In order to investigate the underlying mechanisms associated with differences in secondary cell wall biosynthesis between the two *M. ruthenica* cultivars, genome-wide transcription analysis using high-throughput RNA sequencing was performed. The results indicated that 684 unigenes showed significantly differential expression in the two cultivars. Among them, 228 unigenes were preferentially expressed in cv. Zhilixing, whereas 456 unigenes were highly expressed in cv. Mengnong No.1 (Figure 3A and Supplementary Table 3). Based on the above findings on secondary cell wall composition determination in the two *M. ruthenica* cultivars, we reasoned that differences in genes expression related to cellulose and lignin biosynthesis may lead to the different stem types of these *M. ruthenica* cultivars. Therefore, we classified DEGs into functional categories according to GO (Supplementary Table 4), focusing on the DEGs clustered in a series of processes associated with cell wall-related biogenesis (GO:0071554, GO:0071555, GO:0005618, GO:0009832, and GO:0009834 etc.), especially in the biosynthetic processes of lignin (GO:0009809) and cellulose (GO:0016760, GO:0016759, GO:0030244). Interestingly, the genes involved in lignin monomer biosynthetic processes did not show significant expression differences between the two cultivars. However, *Laccase 17* (*LAC17*), which is essential for lignin polymerization, was highly expressed in cv. Zhilixing. A closer examination of the DEGs *Cellulose synthase A catalytic subunit* (*CESA8*) and *Cellulose synthase-like protein E1* (*CSLE1*) related to cellulose synthesis found that they were preferentially expressed in cv. Zhilixing (Figure 3B). These DEGs were further confirmed using qRT-PCR. As a result, the qRT-PCR analyses for cellulose-related genes were not significantly different between the two *M*. *ruthenica* cultivars. By contrast, the expression of *LAC17* in cv. Zhilixing was nearly eightfold higher than that of Mengnong No.1 (Figure 3C).

The DEGs were analyzed by KEGG pathway to further gain additional information about the potential involvement of specific metabolic pathways in the stem-type regulation of *M*. *ruthenica*. A total of 204 DEGs were assigned to KEGG pathways (Supplementary Table 5), in which phenylpropanoid biosynthesis (ko00940) was greatly altered and *LAC17* (TRINITY_DN16669_c0_g2) involved in lignin polymerization showed increased expression in cv. Zhilixing (Figure 3D). The GO and KEGG enrichment analyses indicated that lignin metabolism plays a key role in the different stem types of *M. ruthenica*.

### Identification of differentially expressed genes involved in lignin biosynthesis between the two cultivars

**FIGURE 4:**
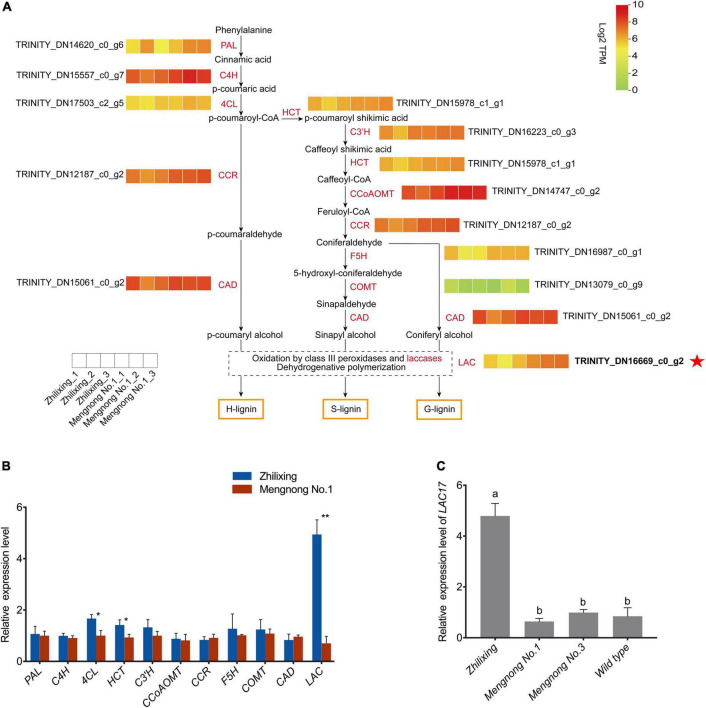
Identification of key gene expression in the lignin synthesis pathway of erect and prostrate stems of *M. ruthenica*. **(A)** Heat map showing genes expression profiles in the lignin biosynthesis pathway. For each heatmap, each small box represents the expression level of a biological sample at the transcription level with TPM values increasing from green to red. Each cultivar is represented by three biological replicates. **(B)** Expression of lignin biosynthesis related unigenes of the two *M. ruthenica* cultivars by PCR analysis. The gene ID is the same as that shown in the heatmap. The expression level in Mengnong No.1 was set to 1. *MrActin* was used as the internal control. Values are means ± S.D. of three biological replicates. Asterisks indicate significant differences determined by Student’s *t*-tests (**p < 0.01, *P < 0.05). **(C)** Expression of monolignol polymerization gene *LAC17* in the erect and prostrate stems. Different lowercase letters indicate highly significant differences (*p* < 0.01).

Lignin biosynthesis pathway involves a series of enzymes, including phenylalanine ammonia lyase (PAL), cinnamic acid 4-hydroxylase (C4H), 4-coumarate-CoA ligase (4CL), cinnamoyl-CoA reductase (CCR), cinnamyl alcohol dehydrogenase (CAD), hydroxycinnamoyl CoA:shikimate hydroxycinnamoyl transferase (HCT), *p*-coumaroyl shikimate 3′-hydroxylase (C3′H), ferulate 5-hydroxylase (F5H), caffeic acid O-methyltransferase (COMT), caffeoyl-CoA 3-O-methyltransferase (CCoAOMT), and laccase (LAC) (Bonawitz and Chapple, 2010; Vanholme et al., 2010). As shown in Figure 4A, we constructed a heat map of the expression levels of lignin-related genes, which indicates that *LAC17* showed significantly different transcript levels, while other lignin biosynthesis genes showed very similar transcript levels between the two cultivars. The RNA-Seq data were also analyzed with the newly published *M. ruthenica* reference genome, and the expression levels of 11 genes in the lignin biosynthesis pathway were consistent with the results of non-reference genome analysis (Supplementary Figure 1; Wang et al., 2021). As shown in Figure 4B, qRT-PCR results of most lignin-genes were consistent with those of the transcriptomic analysis, confirming that the expression level of putative *LAC17* in cv. Zhilixing was significantly higher than that in cv. Mengnong No.1. 4CL and HCT of two cultivars were not identified as DEGs in the RNA-Seq (Figure 4A), but there was a significant difference in the qRT-PCR (*P* < 0.05). Previous molecular and genetic studies have revealed that MYB transcription factors (TFs) are key regulators of the biosynthesis of all three major secondary wall components, including cellulose, xylan, and lignin (Zhong and Ye, 2007; Zhou et al., 2009; Kim et al., 2014; Geng et al., 2019). We also found no significant difference in the expression levels of the MYB TFs regulating lignin biosynthesis between the two cultivars, including *MYB63*, *MYB46, MYB83*, and *MYB20* (Supplementary Figure 2). Overall, the differences in expression levels of monolignol polymerization *LAC17* may explain the difference in terms of lignin content between the two cultivars of *M. ruthenica*. It is well known that most wild-type *M. ruthenica* are prostrate stems (Campbell et al., 1997). Therefore, we selected two other *M. ruthenica* accessions for qRT-PCR verification analysis to further determine the expression level of *LAC17*. Results also showed that the expression of *LAC17* was down-regulated in other prostrate stem accessions (Figure 4C).

### Functional validation of the monolignol polymerization gene *MrLAC17*

**FIGURE 5:**
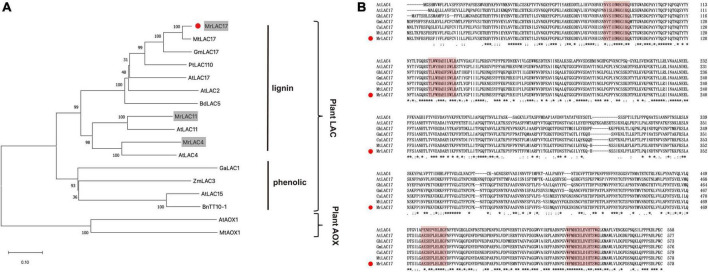
Amino acid sequence alignment and phylogenetic tree between MrLAC17 and other species laccases. **(A)** The distance matrix was clustered using the neighbor-joining method in MEGA software. Bootstrapping set to 1,000 replicates. *M. ruthenica* putative laccases are shown on gray background, and MrLAC17 is marked with red dots. The tree is rooted by the ascorbate oxidase sequences as outgroup. Bar indicates relative branch length. **(B)** Alignment was carried out between MrLAC17 protein and other laccases protein sequences using Clustal Omega, and optimized manually. Numbers indicate the position of the last amino acid in each line of the proteins. The * identical amino acids; or, similar amino acids and **, ***, ****, *****, ******, and ******* indicated that consecutive amino acids were identical (or similar). The copper-binding domains are shaded in red color, and MrLAC17 is marked with red dots.

We constructed a phylogenetic tree with the MrLAC17 amino acid sequences and other plant putative laccases. Specifically, MrLAC17 was clustered in monolignol laccases previously reported to be involved in lignin biosynthesis, including AtLAC17, AtLAC4, PtLAC110, BdLAC5, etc. (Ranocha et al., 2002; Berthet et al., 2011; Wang et al., 2015). AtLAC15, BnTT10-1, GaLAC1, and ZmLAC3 have been previously reported to be involved in polymerization of phenolic compounds (Wang et al., 2004; Caparrós-Ruiz et al., 2006; Liang et al., 2006; Zhang K. et al., 2013; Figure 5A). As predicted by the SignalP server, MrLAC17 had a secretable N-terminal signal peptide, indicating that the protein can be targeted to the cell wall through the endoplasmic reticulum-Golgi network process. In addition, according to the typical characteristics of plant laccases, MrLAC17 exhibited four highly conserved copper-binding domains (Figure 5B).

**FIGURE 6:**
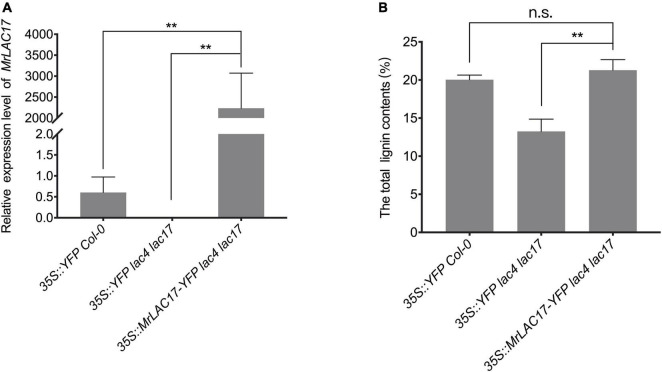
MrLAC17 can complement the *Arabidopsis lac4 lac17* mutant. **(A)** qRT-PCR analysis of MrLAC17 in *Arabidopsis lac4 lac17* mutant complement, *lac4 lac17* double mutants with the empty vector and Col-0 with the empty vector. The expression level in Col-0 was set to 1. *AtActin* was used as the internal control. **(B)** Lignin content in stems of *Arabidopsis lac4 lac17* mutant complement, *lac4 lac17* double mutants with the empty vector and Col-0 with the empty vector. In **(A,B)**, values are means ± S.D. of three technical replicates from three biological replicates. Asterisks indicate significant differences between the two indicated samples, as determined by Student’s *t*-test (**p < 0.01).

To confirm that the *MrLAC17* gene is indeed involved in lignin polymerization, we overexpressed *MrLAC17* in *Arabidopsis lac4 lac17* double mutants. The *MrLAC17* expression levels in several independent transformants were further confirmed by qRT-PCR analysis. Here, we present results for the three top-performing lines (*p* < 0.01, Figure 6A). The *lac4 lac17* double mutants had no growth phenotype (Berthet et al., 2011; Zhao et al., 2013), nor did overexpressed *MrLAC17* exhibit an altered phenotype (Supplementary Figure 3). The total lignin content of 4-week-old plant stems was determined by acetyl bromide method to verify the effect of MrLAC17 overexpression on lignin biosynthesis As shown in Figure 6B, the total lignin contents of overexpressed *MrLAC17* were 21.30%, which was significantly higher than that in *Arabidopsis lac4 lac17* double mutants with the empty vector (13.25%). As expected, the acetyl bromide lignin level of the overexpressed *MrLAC17* was similar to that of wild-type plants (*p* < 0.01, Figure 6B).

### Transcriptional regulation does not explain the difference in *lac17* transcript level between the two cultivars

In order to investigate the mechanism underlying differences in *LAC17* expression, the 1,737 bp open reading frame of *MrLAC17* was cloned from the two cultivars. There was only one silent point mutation between Zhilixing and Mengnong No.1, which did not result in a change in the protein amino acid sequence (Supplementary Figure 4).

The 1.0 kb promoter of *MrLAC17* was further cloned from two *M. ruthenica* cultivars, and their *cis*-acting elements were predicted using PLACE (Supplementary Figure 5B; Higo et al., 1999). It has been well-documented that most lignin biosynthetic genes contain the so-called AC elements, including AC-I (ACCTACC), AC-II (ACCAACC), and AC-III (ACCTAAC) in the promoters that are recognized by MYB TFs (Raes et al., 2003; Rogers and Campbell, 2004). In accordance with the cloned promoter sequence alignment and prediction analysis, no mutation was found in the putative binding sites for MYB TFs between the two *M. ruthenica* cultivars (Supplementary Figures 5A,B). To further verify whether *MrLAC17* is regulated by lignin biosynthesis TFs in both cultivars, the dual-luciferase assay was used to test the transcription factor MYB on activation of a reporter gene, firefly luciferase, driven by the promoter of *MrLAC17*. AtMYB63 is a transcriptional regulator specifically activating lignin biosynthetic genes, including *LAC17* (Zhou et al., 2009). Activation of the *MrLAC17* promoters by heterologous AtMYB63 TF was further analyzed using the transient expression assays in *N. benthamiana* leaves, whereas the promoter activation efficiency of *MrLAC17* was similar between the two cultivars (Supplementary Figure 5C).

### *In vivo* expression analysis indicated that miR397a is less expressed in erect stems of *Medicago ruthenica*

**FIGURE 7:**
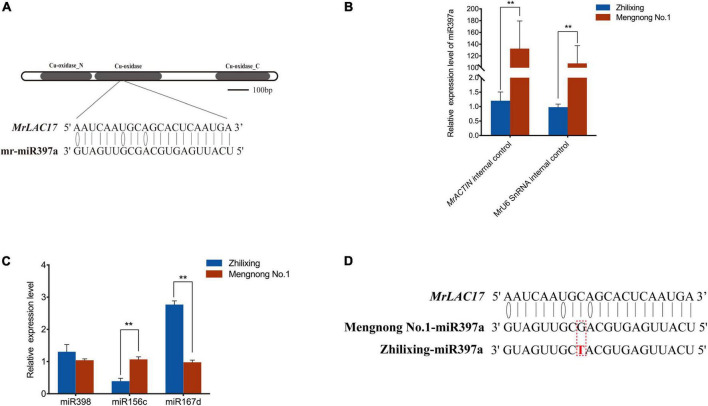
miR397a was specifically low-expressed in the erect stem cultivar of *M. ruthenica*. **(A)** mr-miR397a targets *MrLAC17* for cleavage. **(B)** miRNA-specific qRT-PCR analysis of miR397a in the two *M. ruthenica* cultivars. Expression level of cultivar Zhilixing was set to 1. *MrACTIN and* MrU6 SnRNA were used as the internal control. **(C)** Relative expression results of miRNAs in the two *M. ruthenica* cultivars. The expression level in Mengnong No.1 was set to 1. *MrACTIN* was used as the internal control. In **(B,C)**, values are means ± S.D. of three technical replicates from six biological replicates. Asterisks indicate significant differences between the two indicated samples, as determined by Student’s *t*-test (**p < 0.01). **(D)** A single base mutation in miR397a of the erect stem cultivar. The mutated nucleotide is marked in red.

miRNAs are endogenous small non-coding RNAs involved in regulating gene expression at the post-transcriptional level (Axtell et al., 2007). miR397 are predicted or have been validated to target plant laccase, and *LAC2*, *LAC4*, and *LAC17* mRNAs have been reported as targets of miR397 (Abdel-Ghany and Pilon, 2008; Li et al., 2010; Lu et al., 2013; Wang et al., 2014). To determine whether *MrLAC17* is regulated by miRNAs, we constructed small RNA databases of two *M. ruthenica* cultivars, and screened target miRNAs for *MrLAC17* mRNA using TargetFinder and psRobot. The result showed that mr-miR397a was the only miRNA sequences targeting *MrLAC17*, which encodes a conserved Cu-oxidase domain (Figure 7A). Expression analysis of the miR397a in stems by stem-loop qRT-PCR using specific primers showed highly expressed of *miR397a* in cv. Mengnong No.1, but expression abundance was very low in cv. Zhilixing (Figure 7B). We also undertook expression analysis of other miRNAs, including miR398, miR167d and miR156c. The results showed that the dicer shear system was normal and miR397a was specifically regulated (Figure 7C).

**FIGURE 8:**
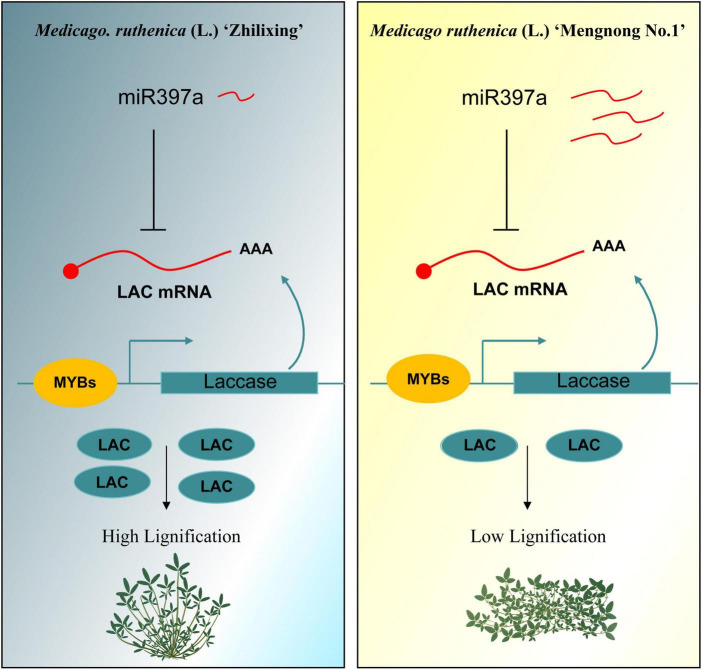
The miR397a-*LAC17* module regulates lignin content in *M. ruthenica* erect and prostrate stems. In the *M. ruthenica* cultivar Zhilixing (diagram on the left), miR397a is maintained at a low expression level, and negatively regulated *LAC17* to a high expression level, thereby leading to enhanced lignin content and an erect-stem phenotype. In the right diagram, the cultivar Mengnong No.1 showed a miR397a-*LAC17* module with the opposite expression level to Zhilixing.

To further explain possible reasons for the differential expression in *miR397a*, we focus on comparing the miRNAs of the two varieties. Interestingly, the sequence of miR397a was not found in multiple samples of cv. Zhilixing. Finally, the alignment was performed under the condition of allowing 1–2 base mismatches, and one-base mutation was found in miR397a of cv. Zhilixing (Figure 7D). These results suggested that miR397a-mediated regulation of *MrLAC17* enhances lignin content, thereby leading to the erect-stem phenotype (Figure 8).

## Discussion

Stem mechanical strength has always been considered to be an important agronomic trait in forage crops. Forage species with high stem mechanical strength have a greater ability to adapt to a wide range of abiotic and biotic habitat conditions, and afford advantages for mechanical harvesting and utilization (Carpita, 1996). Stem diameter and plant height have been reported as key morphological factors affecting stem mechanical strength in crops (Okuno et al., 2014; Zhang et al., 2016; Kamran et al., 2018). Similar results were found in *M. ruthenica*, since the stem diameter and plant height of the erect cultivar were significantly higher than those of prostrate cultivar, indicating that stem straightness was significant positively correlated with its diameter and plant height.

The microstructure of plant stems can also be used as an indicator to estimate their mechanical strength (Kong et al., 2013). The xylem area and percentage in stem anatomy have been found to be positively correlated with stem strength (Mangieri et al., 2016; Liu et al., 2019). Furthermore, lignin, which plays an important role in the formation of the secondary cell wall, is cross-linked with cellulose and hemicellulose to enhance the rigidity and strength the cell walls of plant stems (Vanholme et al., 2010). Abundant studies on stem characteristics of rice, maize, buckwheat, barley, and other crops have shown that lignin is significantly positively correlated with stem mechanical strength, which determines the lodging resistance of plants (Chen et al., 2011; Kong et al., 2013; Begović et al., 2018; Liu et al., 2018). In this study, the lignin content in stems of the erect *M. ruthenica* cultivar was significantly higher than that in the prostrate cultivar, which confirms the relevance of the high lignification observed in the stem anatomy of erect cultivars. Therefore, the erect-type stem might be attributed to the high lignin content of *M. ruthenica* stem.

It is well known that lignin synthesis is a comprehensive reflection of regulatory processes of lignin-related gene expression (Jinmi et al., 2015). In this study, we found that *LAC17* expression level in the erect-type stem was significantly higher than that in the prostrate stem cultivar, while other genes activities showed no clear differences in the lignin biosynthetic pathway. Since 1959, evidence has implicated laccases in lignin polymerization (Freudenberg, 1959). In a previously reported publication of *Arabidopsis thaliana*, the *lac4 lac17* double mutant reduced lignin content by as much as 40%, but the reduction in lignin levels in each single mutant was not significant, providing clear genetic evidence for the function of laccases (Berthet et al., 2011). Although metabolite determination and lignin-related genes expression analysis of can be linked to the process of lignin synthesis, transgenic validation is more reliable in elucidating gene function. To construct efficient tissue culture and plant regeneration system are the prerequisites for genetic transformation of *M. ruthenica*. At present, the research on genetic transformation of *M. ruthenica* is in a bottleneck stage, and needs to be further improved. Therefore, to provide genetic evidence for the involvement of *MrLAC17* in lignin biosynthesis, the *Arabidopsis lac4 lac17* double mutant was genetically complemented with the CDS of *MrLAC17*. The overexpression of *MrLAC17* in the *lac4 lac17* background resulted in a complete recovery of lignin content to the wild-type level.

The above results confirmed that *MrLAC17* is involved in lignin synthesis, but the regulatory mechanism of different *MrLAC17* expression levels between the two cultivars remained unclear. Firstly, we cloned the *MrLAC17* gene in two *M. ruthenica* cultivars, respectively, and no point mutation was found. We further proposed that the expression differences of *MrLAC17* between the two cultivars may be caused by differences in TFs at the transcriptional level, or in mr-miRNAs at the post-transcriptional level. The putative interaction of *MrLAC17* with TFs can be inferred from different *cis*-elements in the *MrLAC17* promoter sequences. Previous studies have shown that the promoters of most lignin-related genes include AC elements, which are identified by MYB TFs (Raes et al., 2003; Rogers and Campbell, 2004). It has been reported that MYB58 was able to bind to AC elements and directly activate the expression of laccase gene (*LAC4*) in *Arabidopsis* (Zhou et al., 2009). No mutation was found in the AC *cis*-elements between the two *M. ruthenica* cultivars by sequence alignment and prediction analysis of the cloned *MrLAC17* promoter. In addition, previous molecular and genetic studies have revealed that MYB TFs are key regulators of lignin biosynthesis, including *MYB63*, *MYB46, MYB83*, and *MYB20* (Zhong and Ye, 2007; Zhou et al., 2009; Kim et al., 2014; Geng et al., 2019). The expression levels of the aforementioned MYB TFs were not significantly affected between the two cultivars.

It has been reported that miRNA can negatively regulate laccase expression by degrading target mRNA in flowering plants. miR408, miR397, and miR857 were validated to target the laccase transcripts in *Arabidopsis* (Abdel-Ghany and Pilon, 2008). Similarly, Ptr-miR397a and Os-miR397 were identified as negative regulators of *PtrLACs* and *OsLAC* in *P. trichocarpa* and *Oryza Sativa*, respectively (Li et al., 2010; Lu et al., 2013). These findings together suggest a potential strategy to indirectly modify laccase expression by regulating miRNAs expression. In the present study, *MrLAC17* was predicted to be a mr-miR397a target. Expression analysis of the miR397a in the stems using stem-loop qRT-PCR showed highly expressed of *miR397a* in the prostrate-type stem, whereas expression abundance was very low in the erect-type stem. Mutations in miRNA-binding sites have been reported to affect transcript cleavage, resulting in altered target gene expression, which affects plant phenotype (Zhang et al., 2017; Jiang et al., 2022). Our research result is that the mutation point is at a relatively conservative mature sequence site in miR397a, which provides a new inspiration for miRNA regulation.

## Conclusion

In conclusion, the functional characterization of the *MrLAC17* in *M. ruthenica* was reported for the first time in this paper. Our study revealed that the miR397a-*LAC17* module regulates lignin content in *M. ruthenica*. The low expression level of miR397a resulted in an decreased expression level of *MrLAC17* and then enhanced lignin content and showed an erect-stem phenotype. The prostrate-stem cultivar showed a miR397a-*LAC17* module with the opposite expression level to erect-stem cultivar (Figure 8). Our work contributes to knowledge to inform strategies for molecular genetic breeding of *M. ruthenica*, and suggests a possible strategy for breeding erect stem cultivars.

## Data availability statement

The original contributions presented in this study are publicly available. This data can be found here: NCBI, PRJNA843576.

## Author contributions

FS, QZ, and YZ conceived and designed the study. YZ and XS conducted the experiments. YZ wrote the manuscript. FS and QZ revised the manuscript. All authors carried out the data analysis and contributed to the article and approved the submitted version.

## Footnotes

1
https://www.ncbi.nlm.nih.gov/genome/?term=WNNG00000000

2
http://www.geneontology.org

3
https://www.genome.jp/kegg/

4
http://www.mirbase.org/

5
https://www.ncbi.nlm.nih.gov/tools/primer-blast/

6
http://bioinformatics.psb.ugent.be/webtools/plantcare/html/

7
https://www.dna.affrc.go.jp/PLACE/?action=newplace

8
http://www.ebi.ac.uk/Tools/msa/clustalo/
