# Supplementary material for: The MicroRNA397a-LACCASE17 module regulates lignin biosynthesis in Medicago ruthenica (L.)
Source: Front Plant Sci. 2022 Aug 18;13:978515. doi: 10.3389/fpls.2022.978515 (PMC9434696; doi:10.3389/fpls.2022.978515)
Supplement: Supplementary file 1 [file Data_Sheet_1.PDF]

Supplementary Material 1

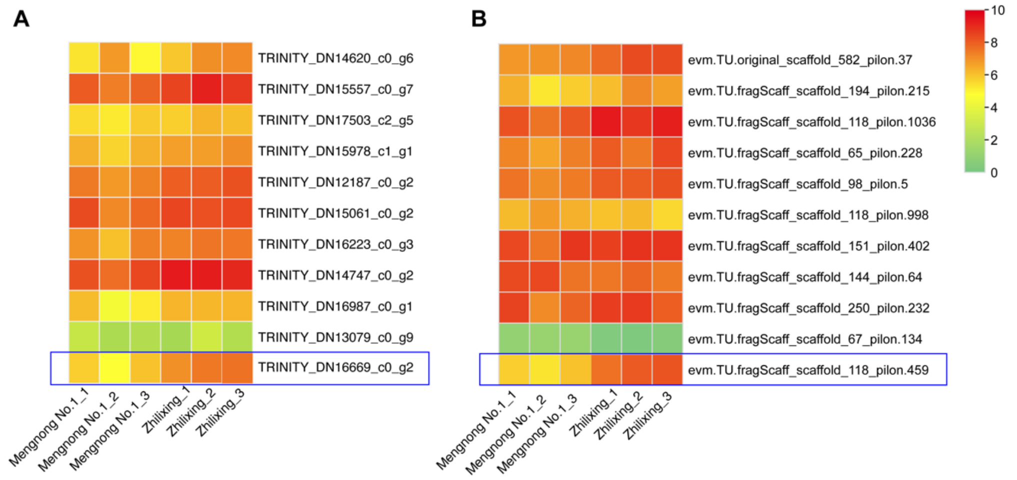

Supplementary Figure 1. Heat maps of the expression pattern of lignin synthesis-related genes with different analysis methods of transcriptome data in the *M. ruthenica* cultivars Zhilixing and Mengnong No.1.

(A) Heat map of the expression pattern of lignin synthesis-related genes by non-reference genome analysis.

(B) Heat map of the expression pattern of lignin synthesis-related genes by reference genome analysis.

In **(A)** and **(B)**, the gene IDs from top to bottom are *PAL*, *C4H*, *4CL*, *HCT*, *CCR*, *CAD*, *C3'H*, *CoAOMT*, *F5H*, *COMT* and *LAC*, respectively. For each heatmap, each small box represents the expression level of a biological sample at the transcription level with TPM values increasing from green to red. Each cultivar is represented by three biological replicates.

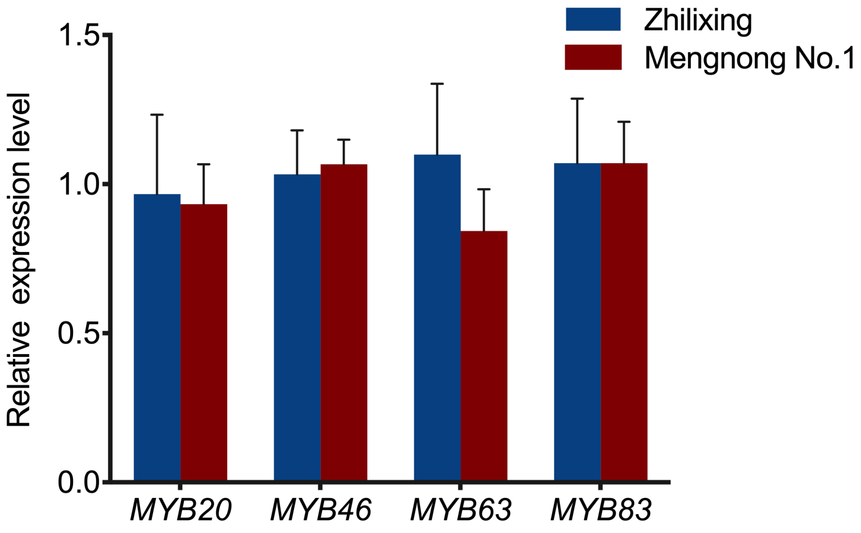

Supplementary Figure 2. Expression of MYB transcription factors regulating lignin biosynthesis in the *M. ruthenica* cultivars Zhilixing and Mengnong No.1 by qRT-PCR analysis.

The expression level in Mengnong No.1 was set to 1. *MrActin* was used as the internal control. Values are means ± S.D. of three biological repeats.

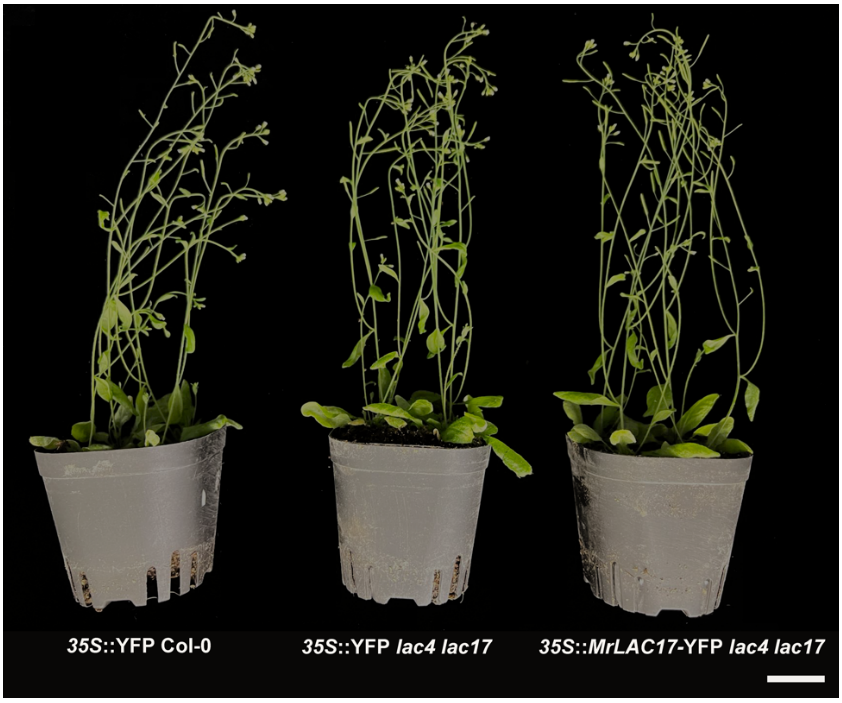

Supplementary Figure 3. No altered phenotype was observed with *MrLAC17* overexpressed in the *lac4 lac17* double mutants of *Arabidopsis*. Bar = 2 cm.

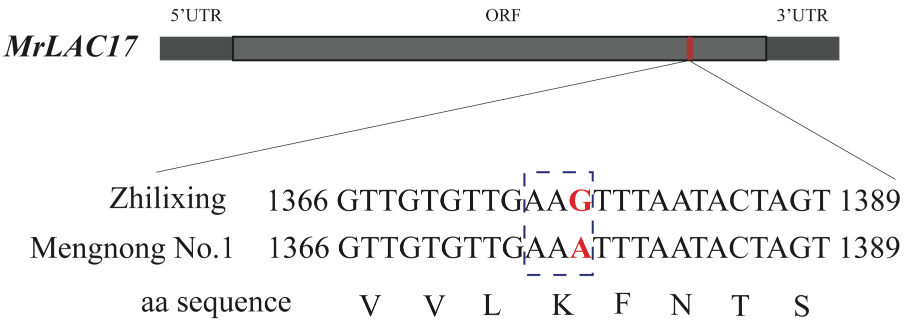

Supplementary Figure 4. *MrLAC17* construct in pEASY-Blunt Zero cloning vector: breakout showing a silent point mutation between *MrLAC17* CDS sequences of the *M. ruthenica* cultivars Zhilixing and Mengnong No.1.

Six biological replicates of each cultivar were cloned and verified.

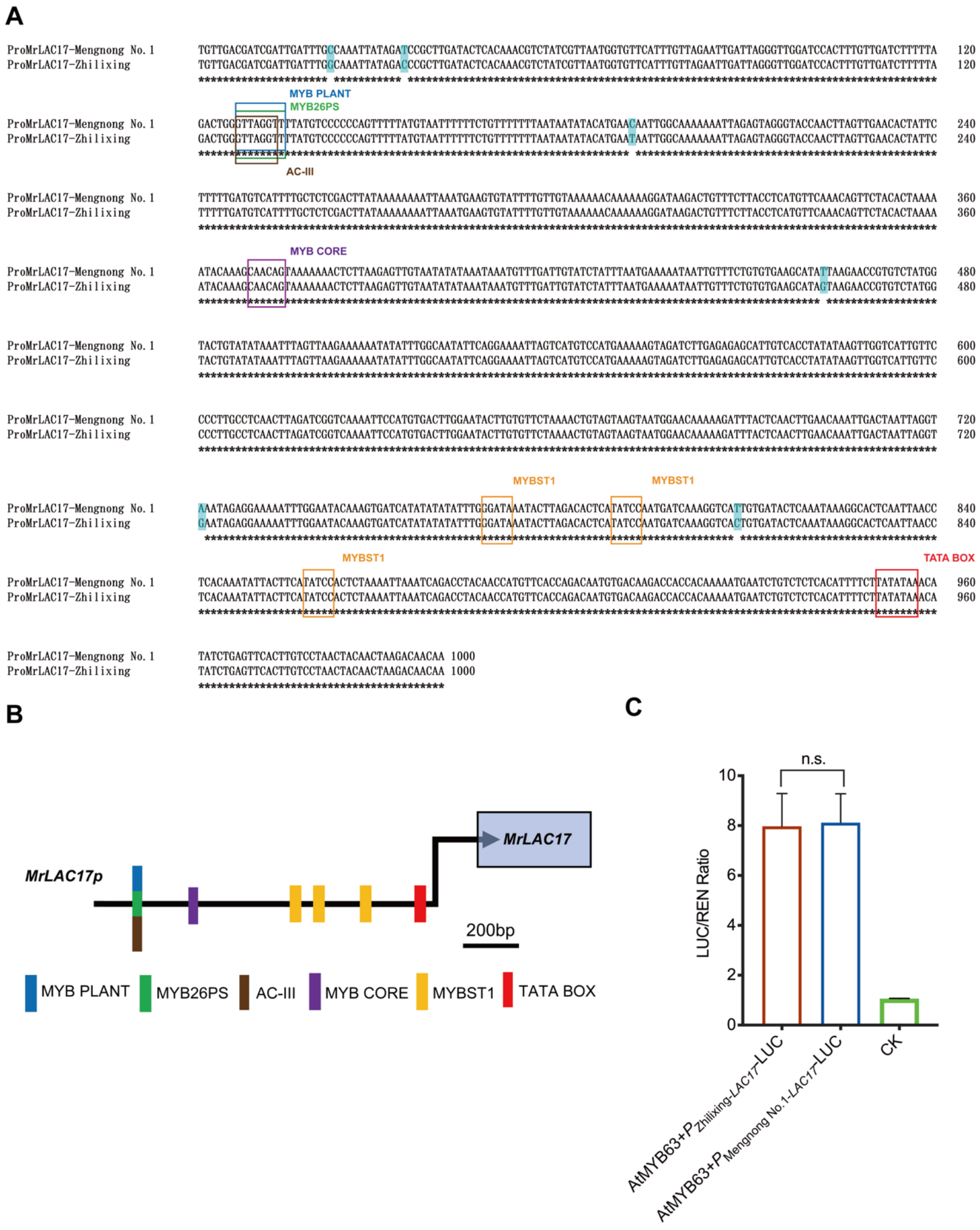

Supplementary Figure 5. Identification and validation of *MrLAC17* promoters in the two *M. ruthenica* cultivars Zhilixing and Mengnong No.1.

(A) *MrLAC17* promoter sequence alignment between the two *M. ruthenica* cultivars Zhilixing and Mengnong No.1. ∗, identical base; blue colored area, different bases. The different colored rectangular marquee corresponds to the putative *cis* elements in **Supplementary Figure 5B.**

(B) Schematic representation of putative cis elements in *MrLAC17* promoter sequences.

The transcription start site is shown by an arrow.

(C) Transient expression assays showed that the promoters of both Zhilixing and Mengnong No.1 *MrLAC17*($P_{\text{Zhilixing-}\mathit{LAC17}}$ and $P_{\text{Mengnong.No.1-}\mathit{LAC17}}$) can be activated by AtMYB63. The $P_{\text{Zhilixing-}\mathit{LAC17}}$-LUC and $P_{\text{Mengnong.No.1-}\mathit{LAC17}}$-LUC reporters were cotransformed with the indicated constructs in *N. benthamiana* leaves. The LUC/REN ratio represents the LUC activity relative to the REN activity. CK was the vector of GUS-pEarleyGate 101, used as a control, and was set to 1. Data are means ± SD of three biological replicates.

Supplementary Table 1. Primers used in this study

Primer name	Sequence (5' to 3')	Function
MrACTIN-qRT -F	ATCCAGGCTGTCCTCTCCCT	qRT-PCR
MrACTIN-qRT -R	ACGAAGGATGGCATGTGGGA	qRT-PCR
AtACTIN-qRT -F	GGTAACATTGTGCTCAGTGGTGG	qRT-PCR
AtACTIN-qRT -R	AACGACCTTAATCTTCATGCTGC	qRT-PCR
LAC17-qRT -F	GGATTGAGAATGGCATGGCTTGTAT	qRT-PCR
LAC17-qRT -R	TGGAAGATCAGAAGGTGGTGGTT	qRT-PCR
CESA8-qRT -F	AATTCACGCGACCATCCTGG	qRT-PCR
CESA8-qRT -R	CAGCACCAGCCTTTTTGTGG	qRT-PCR
CSLE1-qRT -F	CGCCAAGCCCTTAGATGGAA	qRT-PCR
CSLE1-qRT -R	TCTCCGGATTTGCCGTACAC	qRT-PCR
PAL- qRT -F	GCCGTTGGTTCTGGTTTAGC	qRT-PCR
PAL- qRT -R	GGGTGGTGTTTCAACTTGTGT	qRT-PCR
CCR- qRT -F	AGGGATGTGGCATTAGCTCAC	qRT-PCR
CCR- qRT -R	TTTCAACAAGCTCCCCACGA	qRT-PCR
4CL- qRT -F	GGCACAGTCGTAAGAAACGC	qRT-PCR
4CL- qRT -R	AGTTCTCTTTGTCGCCTCCG	qRT-PCR
C4H- qRT -F	CACTGGAAAAAGCCGGAGGA	qRT-PCR
C4H- qRT -R	TTCTGCCAACACCGAAAGGA	qRT-PCR
HCT- qRT -F	TCTTGTTCGCGGTGCACATA	qRT-PCR
HCT- qRT -R	AATTGGCCTTCCCCAACCAA	qRT-PCR
COMT- qRT -F	TGGAGGTGGTCTTGGGGTTA	qRT-PCR
COMT- qRT -R	ACTCCAGGATATGAAGGGGCA	qRT-PCR

CCoAOMT- qRT -F	TGATCGGCTACGACAACACC	qRT-PCR
CCoAOMT- qRT -R	CCTAGGGTCCACAGCCAAAG	qRT-PCR
CAD- qRT -F	GTTGGTCACCCTCTTGAGCC	qRT-PCR
CAD - qRT -R	GCTCCCAACAAAGCTTCCAG	qRT-PCR
F5H- qRT -F	GAGGAACGGAAACGGTAGCA	qRT-PCR
F5H - qRT -R	GGCCCACAACTTCTGCTAGT	qRT-PCR
C3'H- qRT -F	AGCTATGGCAGAGCACATCC	qRT-PCR
C3'H- qRT -R	GTTTGGCACCACCGGATTTC	qRT-PCR
MYB63- qRT -F	CAACAAGGATGAGGTCCTACCA	qRT-PCR
MYB63- qRT -R	TGAGGCAAAGAGGGCTACAA	qRT-PCR
MYB46- qRT -F	ACACGTTGCCTATGCTGGAT	qRT-PCR
MYB46- qRT -R	ACGACTTTGTGACGAAAAGCA	qRT-PCR
MYB83- qRT -F	CCAACTTTATGGTGCAGCCG	qRT-PCR
MYB83- qRT -R	CAATGCAGCTGGTTGTACCG	qRT-PCR
MYB20- qRT -F	GAAAGGACCATGGACTGCTGA	qRT-PCR
MYB20- qRT -R	ACAGCTCTCCAGCAACATTGA	qRT-PCR
MrLAC17-F	ATGGAGTTGACCAACTTTCATTC	Cloning
MrLAC17-R	TCAACATTTTGGAAGATCAGAAGG	Cloning
1300- MrLAC17-CDS-F	CCGAATTCGGAGTCGACACTAGTATGGAGTTGACCAACTTTCATTC	Plasmid construction
1300- MrLAC17-CDS-R	TCCACCTCCGACCGGTGCACTAGTACATTTTGGAAGATCAGAAGGTG	Plasmid construction
MrproLAC17-SP1-R	GCGGCTGCAAGACCTCAATTTTCTAT	Cloning
MrproLAC17-SP2-R	CACTAAGAATGGTTCTTGTGTGGCAC	Cloning
MrproLAC17-SP3-R	CTGCTCACTACAAGCTCAGGAAGCA	Cloning

*Mr*proLAC17-HindIII-F	CGGTATCGATAAGCTTGTTATATCTACTGTTGATCCTCACA	Plasmid construction
*Mr*proLAC17-PstI-R	CGGGCTGCAGGAATTCTTGTTGTCTTAGTTGTAGTTAGGAC	Plasmid construction
TOPO-AtMYB63-F	CACCATGGGGAAGGGAAGAGCACC	Plasmid construction
TOPO-AtMYB63-R	ATGTATCATGAGCTCGTAGT	Plasmid construction
U6 snRNA-F	ACAGAGAAGATTAGCATGGCCC	qRT-PCR
U6 snRNA-R	GACCATTTCTCGATTTGTGCG	qRT-PCR
Stem loop miR397a-RT	GTCGTATCCAGTGCAGGGTCCGAGGTATTCGCACTGGATACGACCATCAA	Stem loop -RT primer
mi397a-qRT-F	GGCTCATTGAGTGCAGCG	qRT-PCR
mi397a-qRT-R	ATCCAGTGCAGGGTCCGAGG	qRT-PCR
Stem loop miR398-RT	GTCGTATCCAGTGCAGGGTCCGAGGTATTCGCACTGGATACGACAGGGGT	Stem loop -RT primer
mi398-qRT-F	GGCTGTGTTCTCAGGTC	qRT-PCR
mi398-qRT-R	ATCCAGTGCAGGGTCCGAGG	qRT-PCR
Stem loop miR167d-RT	GTCGTATCCAGTGCAGGGTCCGAGGTATTCGCACTGGATACGACCCAGAT	Stem loop -RT primer
mi167d-qRT-F	GGCTGAAGCTGCCAGCATG	qRT-PCR
mi167d-qRT-R	ATCCAGTGCAGGGTCCGAGG	qRT-PCR
Stem loop miR156c-RT	GTCGTATCCAGTGCAGGGTCCGAGGTATTCGCACTGGATACGACGTGCTC	Stem loop -RT primer
mi156c-qRT-F	GGCTTGACAGAAGAGAGA	qRT-PCR
mi156c-qRT-R	ATCCAGTGCAGGGTCCGAGG	qRT-PCR

Supplementary Table 2. Database accession numbers of plant laccases (ascorbate oxidase)

Plant	Laccase（ascorbate oxidase ）	Accession number
Arabidopsis thaliana	AtLAC2	[TAIR: AT2G29130]
	AtLAC4	[TAIR: AT2G38080]
	AtLAC11	[TAIR: AT5G03260]
	AtLAC15	[TAIR: AT5G48100]
	AtLAC17<br>AtAOX1	[TAIR: AT5G60020]<br>[TAIR:AT4G39830]
Brachypodium distachyon	BdLAC5	[Phytozome: Bradi1g66720]
Brassica napus	BnTT10-1	[Genbank: AEK27149]
Gossypium arboreum	GaLAC1	[Genbank: AAR83118]
Populus trichocarpa	PtLAC110	[EMBL: CAA74105]
Saccharum officinarum	SofLAC	[SUCEST: SCUTST3084C11.g]
Zea mays	ZmLAC3	[EMBL: CAJ30499]
Glycine max	GmLAC17	[Genbank: XP_003551482]
Medicago truncatula	MtLAC17	[Genbank: XP_013448882]
	MtAOX1	[Genbank: CAA75577]
Medicago ruthenica	MrLAC17	[Genbank: OM371323]
